# Cross-over endocytosis of claudins is mediated by interactions via their extracellular loops

**DOI:** 10.1371/journal.pone.0182106

**Published:** 2017-08-15

**Authors:** Nora Gehne, Agathe Lamik, Martin Lehmann, Reiner F. Haseloff, Anuska V. Andjelkovic, Ingolf E. Blasig

**Affiliations:** 1 Leibniz-Forschungsinstitut für Molekulare Pharmakologie, Berlin, Germany; 2 University of Michigan, Medical School, Ann Arbor, United States of America; Universitatsklinikum Hamburg-Eppendorf, GERMANY

## Abstract

Claudins (Cldns) are transmembrane tight junction (TJ) proteins that paracellularly seal endo- and epithelial barriers by their interactions within the TJs. However, the mechanisms allowing TJ remodeling while maintaining barrier integrity are largely unknown. Cldns and occludin are heterophilically and homophilically cross-over endocytosed into neighboring cells in large, double membrane vesicles. Super-resolution microscopy confirmed the presence of Cldns in these vesicles and revealed a distinct separation of Cldns derived from opposing cells within cross-over endocytosed vesicles. Colocalization of cross-over endocytosed Cldn with the autophagosome markers as well as inhibition of autophagosome biogenesis verified involvement of the autophagosomal pathway. Accordingly, cross-over endocytosed Cldns underwent lysosomal degradation as indicated by lysosome markers. Cross-over endocytosis of Cldn5 depended on clathrin and caveolin pathways but not on dynamin. Cross-over endocytosis also depended on Cldn-Cldn-interactions. Amino acid substitutions in the second extracellular loop of Cldn5 (F147A, Q156E) caused impaired *cis*- and *trans*-interaction, as well as diminished cross-over endocytosis. Moreover, F147A exhibited an increased mobility in the membrane, while Q156E was not as mobile but enhanced the paracellular permeability. In conclusion, the endocytosis of TJ proteins depends on their ability to interact strongly with each other in *cis* and *trans*, and the mobility of Cldns in the membrane is not necessarily an indicator of barrier permeability. TJ-remodeling via cross-over endocytosis represents a general mechanism for the degradation of transmembrane proteins in cell-cell contacts and directly links junctional membrane turnover to autophagy.

## Introduction

Tight junctions (TJs) are mainly formed by claudins (Cldns), transmembrane proteins that play a major role in limiting and regulating paracellular permeation in epithelia and endothelia. Cldns are tetraspanning membrane proteins, with both termini located in the cytoplasm, one intracellular and two extracellular loops (ECLs) [1]. Continuous intercellular TJ strands are formed through homo- and heterophilic interactions, both between Cldns in the opposing membranes (*trans*-interaction) via their extracellular domains [2], and between Cldns within the same membrane (*cis*-interaction). Many Cldns contribute to the tightening of tissue barriers, such as Cldn1 [3] and Cldn5 [4], while other Cldns, e.g. Cldn2 and Cldn7, in addition to their sealing function, form paracellular pores to allow selective ion exchange [5, 6].

Endocytosis of Cldns is essential for the regulation of Cldn content in paracellular barriers and can follow different pathways, depending on Cldn subtype and stimulus. Cldn5 is removed from the TJ in a caveolin-dependent manner in endothelial cells of the blood-brain barrier during stroke [7], as well as in cultured endothelial cells after treatment with the cytokine CC-chemokine ligand 2 [8]. Interferon gamma causes Cldn1 to be displaced from the plasma membrane to early and recycling endosomes after 48 h [9], a process similar to macropinocytosis. Calcium depletion in epithelial T84 cells leads to clathrin-dependent internalization of Cldns 1 and 4 into subapical ring-like organelles that are positive for syntaxin-4, but not for markers of lysosomes, the Golgi apparatus, or late or recycling endosomes [10]. These data indicate that there may be a poorly characterized compartment involved in Cldn internalization. While there is an abundance of information regarding stimuli leading to TJ disruption, there is only limited knowledge on constitutive internalization pathways of TJ proteins.

After internalization, claudins can either be targeted for degradation or for recycling to the plasma membrane. In type II Madin Darby canine kidney cells (MDCK-II), Cldn1 and Cldn2 are continuously recycled, and this depends on a functioning ESCRT complex (endosomal sorting complex required for transport) [11]. Degradation of internalized Cldns has been shown to depend on both lysosomal and proteasomal pathways: in MDCK-I cells, Cldn1 is ubiquitinated and subsequently degraded via the lysosome [12]; human Cldn5 is degraded by the proteasome after poly-ubiquitination at K199, but also by an ubiquitin-independent lysosomal mechanism in endothelial cells, and it has a relatively short half-life of 90 min [13]. This indicates continuous TJ turnover, although the details of this remodeling are unclear.

While TJ-strands are highly mobile in the membrane, the individual Cldns within the strands are not [14]. More than 75% of Cldn1 is immobile within the TJs, as measured by fluorescence recovery after photobleaching (FRAP) in MDCK-I cells [15]. Similarly, Cldn1 transfected into TJ-free human embryonic kidney 293 (HEK-293) cells displays an immobile fraction of approximately 70% [16], which is comparable to Cldn5 [17], and it is assumed that the immobilized fraction contains the Cldn molecules that are bound to neighboring Cldns. This raises the question of how the dynamic behavior of TJs is realized while still maintaining a functionally stable barrier. Removal of Cldns from intact TJs poses a challenge, due to the strong *cis*- and *trans*-interactions occurring within TJ-strands. Recombinant extracellular loops of Cldns have been shown to associate to the extracellular loops of full-length Cldns, with very high affinity in the low nanomolar range [18]. Breaking these interactions would require proteolysis in the extracellular environment, and the inaccessible binding surface of the extracellular loops makes it unlikely that Cldns from opposing cells are separated before endocytosis. Accordingly, Cldns have been indicated to be endocytosed from one cell into neighboring cells [19], however the driving force of this process and the endocytic compartment of the resulting vesicles are not explored.

To clarify the mechanism of endocytosis into the neighboring cell, the contribution of different endocytosis pathways and autophagy were investigated for TJ proteins that contribute to the formation and tightening of important tissue barriers in the organism. Moreover, the role of Cldn-Cldn interactions in this process was elucidated.

## Materials and methods

### Constructs and stable cell lines

Murine Cldn1 or Cldn5 were subcloned into pEYFP-C1 (Clontech) and pmTurquoise2-C1 [20] vectors (Primers: Cldn1-fwd 5’ - CACCAAGCTTCCATGGCCAACGCGGGGCTG; Cldn1-rev 5’ - GCAGGGATCCTCACACATAGTCTTTCCCACTAGA; Cldn5-fwd 5´ - CATCAAGCTTCCATGGGGTCTGCAGCGTTG; Cldn5-rev 5´ - GCAGGGATCCTTAGACATAGTTCTTCTTGTCGTA). The N-terminal position of the fluorophore was chosen to leave the C-terminus free for interaction with the PDZ domain of ZO-1 polymerizing Cldns in the plasma membrane [21], in order to ensure correct localization in the membrane.

MDCK-II [22] or HEK-293 [17] cells were grown in 6-well plates until 80% confluency and transfected using polyethylenimine (PEI; Polysciences Inc, #23966–2) [16]. For each well, 10 μl of PEI (1 mg/ml) were added to 250 μl Opti-MEM (Thermo Fisher Scientific, #31985047) and 2 μg plasmid DNA (YFP-Cldn1, TRQ-Cldn1, YFP-Cldn5 or TRQ-Cldn5) and were mixed with additional 250 μl Opti-MEM. After 5 min incubation at room temperature (RT), the solutions were combined and carefully mixed. Following 25 min incubation at RT, the mixture was added drop wise to each well. Transfection medium was removed after overnight incubation and cells were transferred to a 25 cm^2^ flask. For stable MDCK-II cell lines, cells were treated with G418 500 μg/ml (Biochrom, #A291-25) until cell growth was normal. Stable cell lines were FACS sorted.

### Cell culture and immunocytochemistry

Cells were cultured in DMEM (Thermo Fisher Scientific, #21885025) containing 10% fetal calf serum (Thermo Fisher Scientific, #10270106) and 1% penicillin/streptomycin (Thermo Fischer Scientific, #15140122) at 37°C and 10% CO_2_. For transfected cells, G418 was added to the medium. For immunofluorescence, cells were seeded on glass coverslips coated with poly-L-lysine (PLL; Sigma-Aldrich, #P6282). For analysis of endocytosis, monotransfected MDCK-II cells were grown in YFP-Cldn/TRQ-Cldn cocultures. Inhibitors were added directly to the culture medium: chlorpromazine (50 μM, 1 h; Sigma-Aldrich, #C8138), dynasore (100 μM, 2.5 h; Cayman Chemical, #14062), filipin III (1 μg/ml, 2 h; Cayman Chemical, #70440), chloroquine (100 μM, 3 h; Sigma-Aldrich, #C6628), YM-201636 (1 μM, 3 h; Cayman Chemical, #13576), LY-294002 (100 mM, 2.5 h; Sigma-Aldrich, #L9908), Bafilomycin A1 (100 nM, Cell Signaling Technology, #54645).

After treatment, cells were rinsed twice with ice-cold phosphate buffered saline (PBS; Thermo Fisher Scientific, #14040091) containing Ca^2+^ and Mg^2+^. Fixation was performed on ice by incubation in acetone for 5 min, ethanol for 1 min and rinsing in PBS for 1 min. Cells were blocked with 5% bovine serum albumin (Roth, #8076) in PBS containing 1% TritonX-100 (Roth, #3051) for 15 min at RT. Incubation with primary antibodies was performed in blocking solution at 4°C overnight (anti-ZO-1: 2 μg/ml, Thermo Fisher Scientific, #33–9100; anti-Cldn1: 1 μg/ml, Thermo Fisher Scientific, #51–9000; anti Cldn5: 1 μg/ml, Thermo Fisher Scientific, #34–1600; anti-Lamp1: 5μg/ml, ProSci, #3629; anti-Rab4/11: 1 μl/ml, Sampler Kit, Cell Signaling Technology, #9385S; anti-ATG16L: 5 μg/ml, ProSci, #4425). Coverslips were washed 4 times with PBS for 10 min at RT, followed by incubation with secondary antibody (goat anti-mouse or anti-rabbit IgG AlexaFluor 647 conjugate; Thermo Fisher Scientific, #A-21236 and #A-21244) in blocking solution for 1 h at RT. Excess antibody was removed by washing 4 times with PBS for 10 min at RT and cells were mounted on microscope slides in Immu-Mount (Thermo Fisher Scientific).

### Fluorescence proteinase protection (FPP)-assay

Cells were washed 3x for 1 min in KHM buffer (110 mM potassium acetate, 20 mM HEPES, 2 mM MgCl_2_, pH 7.4). For permeabilization, 100 μM digitonin (Cayman Chemical, #14952) in KHM buffer was applied for 3 min; digestion was carried out using proteinase K (Thermo Fisher Scientific, #EO0491) 300 μg/ml in KHM buffer. All steps were performed at RT.

### Microscopy

To determine vesicle numbers, fixed cells were imaged using a confocal microscope (Zeiss NLO with a Plan-Neofluar 100x 1.3 Oil objective, Zeiss, Oberkochen, Germany). Z-stacks with 0.25 μm intervals between slices through the whole cell were evaluated using ImageJ (http://imagej.nih.gov/ij/). To automatize the vesicle count, an ImageJ macro was created. Fluorescence thresholds were set to 70 for YFP and TRQ and to 40 for Alexa 647. Vesicles with a radius of 50 nm < r < 500 nm were counted in each slice and mean vesicle numbers per slice were calculated. Cross-over endocytosed vesicle numbers were normalized to the total length of the contacts shared with cells expressing the other fluorophore, while total vesicle numbers were normalized to the cell perimeter. For each experiment, vesicle numbers of treated cells were normalized to untreated samples. For live cell imaging, cells were seeded on glass coverslips (r = 15 mm) coated with PLL. Cocultures of monotransfected MDCK-II cells were washed with HBSS+/+ (Hanks balanced salt solution with Ca^2+^ and Mg^2+^; Thermo Fisher Scientific, #14065049) once and imaged in fresh HBSS+/+.

For fluorescence resonance energy transfer (FRET) measurements by photoacceptor bleaching, HEK-293 cells were transiently transfected with TRQ/YFP pairs, as indicated in the figures. After transfection, the cells were grown on glass coverslips for 2 d before measurement. Increase in TRQ-signal was measured after 10 cycles of YFP-bleaching. YFP/TRQ ratios were determined to have a linear effect on FRET-values between ratios 0.5 and 8, only cell-contacts with these ratios were used. FRET efficiencies were normalized to the YFP/TRQ ratio and then to TRQ-Cldn5/YFP-Cldn5.

Fluorescence after photobleaching (FRAP) measurements were performed in cocultures of MDCK-II cells monotransfected with TRQ-Cldn5 or YFP-Cldn5 (and accordingly the F147A or Q156E variants). Before bleaching, 5 pictures were taken of the contact to reach a stable starting intensity. In YFP/TRQ cell-contacts YFP was bleached in an area approx. 10% of the contact length (1 ms pulse, 100% laser intensity) and the signal intensity measured in 5 s intervals for 220 s. Curves were normalized to the range of bleached signal. Mobile fractions were determined by a fitted curve (Graphpad Prism5, http://www.graphpad.com/scientific-software/prism/).

For stimulated emission depletion (STED) images, the Leica TCS 3X gSTED, Leica LASX software and HC PL APO CS2 100x 1.4 Oil objective (Leica, Wetzlar, Germany) were used. TRQ was excited at 405 nm and emission was detected from 440 nm– 513 nm. YFP was excited at 514 nm and emission was collected from 519 nm– 581 nm. Emissions from both proteins were collected on HyD detectors in the time gated mode (0.3–6 ns) in the presence of a 592 nm depletion laser.

### Transepithelial electrical resistance (TER) and paracellular permeation measurements

TER was measured using Cellzscope (nanoAnalytics, Münster, Germany). MDCK-II cells were seeded on hanging PET cell culture inserts (Merck Millipore, #PIHT12R48) at a density of 80,000 cells/insert. After TER values had reached a stable plateau, the cells were transferred to a 24-well plate and washed twice with HBSS+/+ warmed to 37°C. Lucifer yellow (m.w. 457 Da; Sigma-Aldrich, #L0259) was dissolved in warm HBSS+/+ (200 μM) and 200 μl solution were applied to the apical compartment of the insert, in order to determine the permeability coefficient of the cell barrier [23]. The basolateral compartment was filled with 1ml warm HBSS+/+ and the plate was incubated at 37% with 10% CO_2_ for 10 min. The inserts were then moved to a fresh 24-well plate containing prewarmed HBSS+/+ and incubated for two more 10 min periods. From each basolateral compartment, 80 μl of sample were transferred to a 96-well plate in triplicate and Lucifer yellow fluorescence was measured using a Tecan microplate reader (Tecan, Männedorf, Switzerland); concentrations were determined from a standard curve.

### Modeling

Modeling of murine Cldn5 and its mutants was performed using Iterative Threading ASSEmbly Refinement (I-TASSER) [24]. Amino acids 1 to 190 of murine Cldns were included in the modeling (transmembrane domains and extracellular loops, excluding the charged amino acids C-terminally flanking the fourth transmembrane domain) and the models of the mutants were aligned with the wild type model using PyMOL (https://www.pymol.org/).

### Statistics

Data was analyzed using the Kruskal-Wallis test (one way ANOVA) followed by Dunn´s multiple comparison post-test or using a two tailed t test. Statistics and graphs were created using GraphPad Prism5. Further details are given in the figure legends; results are shown as mean±SEM as indicated. If not stated otherwise, differences were considered significant if p<0.05.

## Results

### Claudin oligomers are cross-over endocytosed into neighboring claudin-expressing cells

**Fig 1:**
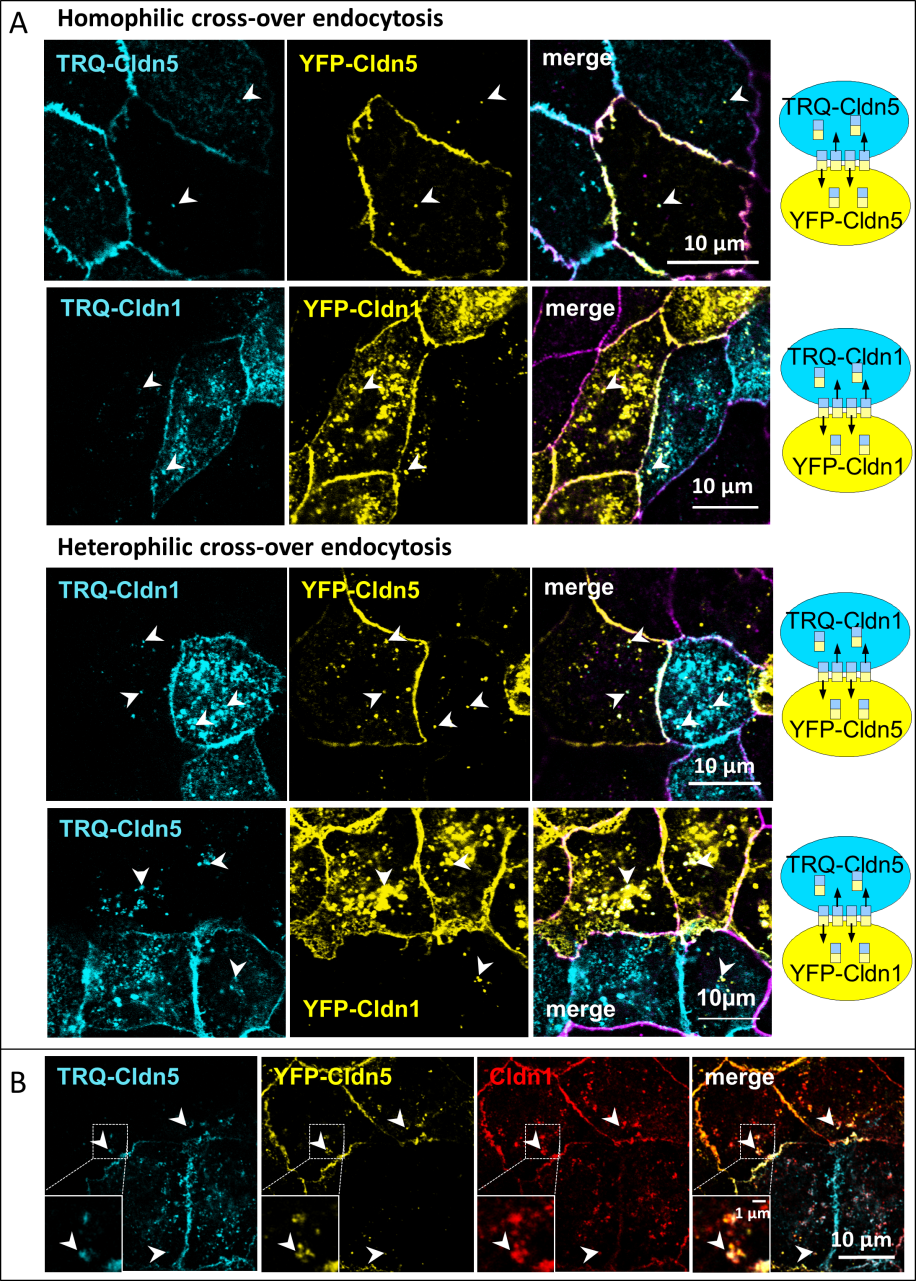
Cross-over endocytosis of claudins (Cldns). Madin-Darby canine kidney II cells expressing a Cldn fluorescently labelled with either yellow fluorescent protein (YFP) or mTurquoise2 (TRQ) were grown in cocultures of monotransfected cells. **A**: Homo- and heterophilic coculture. Arrowheads: vesicles containing cross-over endocytosed Cldns (turquoise or yellow channel). Purple: ZO-1 used as junctional marker protein. On the right: schematic view of cross-over endocytosed Cldn-oligomers. **B**: Endogenous Cldn1 was immunohistochemically stained (red) in cross-over endocytosed Cldn5 vesicles (arrowheads).

Cldn1 and Cldn5 were N-terminally tagged with either mTurquoise2 (TRQ) or yellow fluorescent protein (YFP) and transfected into MDCK-II cells. These were grown in cocultures of cells monotransfected with a YFP- or TRQ-construct. In cells expressing YFP-Cldn sharing a cell contact with TRQ-Cldn expressing cells, cross-over endocytosed vesicles containing both fluorophores were observed in both cells (Fig 1, arrowheads). This effect was found for homophilic cocultures of TRQ-Cldn5/YFP-Cldn5 (Fig 1A homophilic, upper panel) and TRQ-Cldn1/YFP-Cldn1 (Fig 1A homophilic, lower panel), as well as in heterophilic cocultures of TRQ-Cldn1/YFP-Cldn5 (Fig 1A heterophilic, upper panel) and TRQ-Cldn5/YFP-Cldn1 (Fig 1A heterophilic, lower panel). Cross-over endocytosed TRQ-containing vesicles found in YFP-Cldn transfected cells usually contained YFP-Cldn as well. In TRQ-Cldn transfected cells, the converse was found. All the vesicles also contained endogenous TJ proteins, e.g. Cldn1 (Fig 1B), Cldn2, Cldn7 and occludin (S1 Fig). The presence of different TJ-proteins in these vesicles demonstrates that whole sections of the TJs are endocytosed, but not specific Cldn-subtypes.

### Amino acid substitution in extracellular loop 2 of claudins influences their interactions and barrier properties

**Fig 2:**
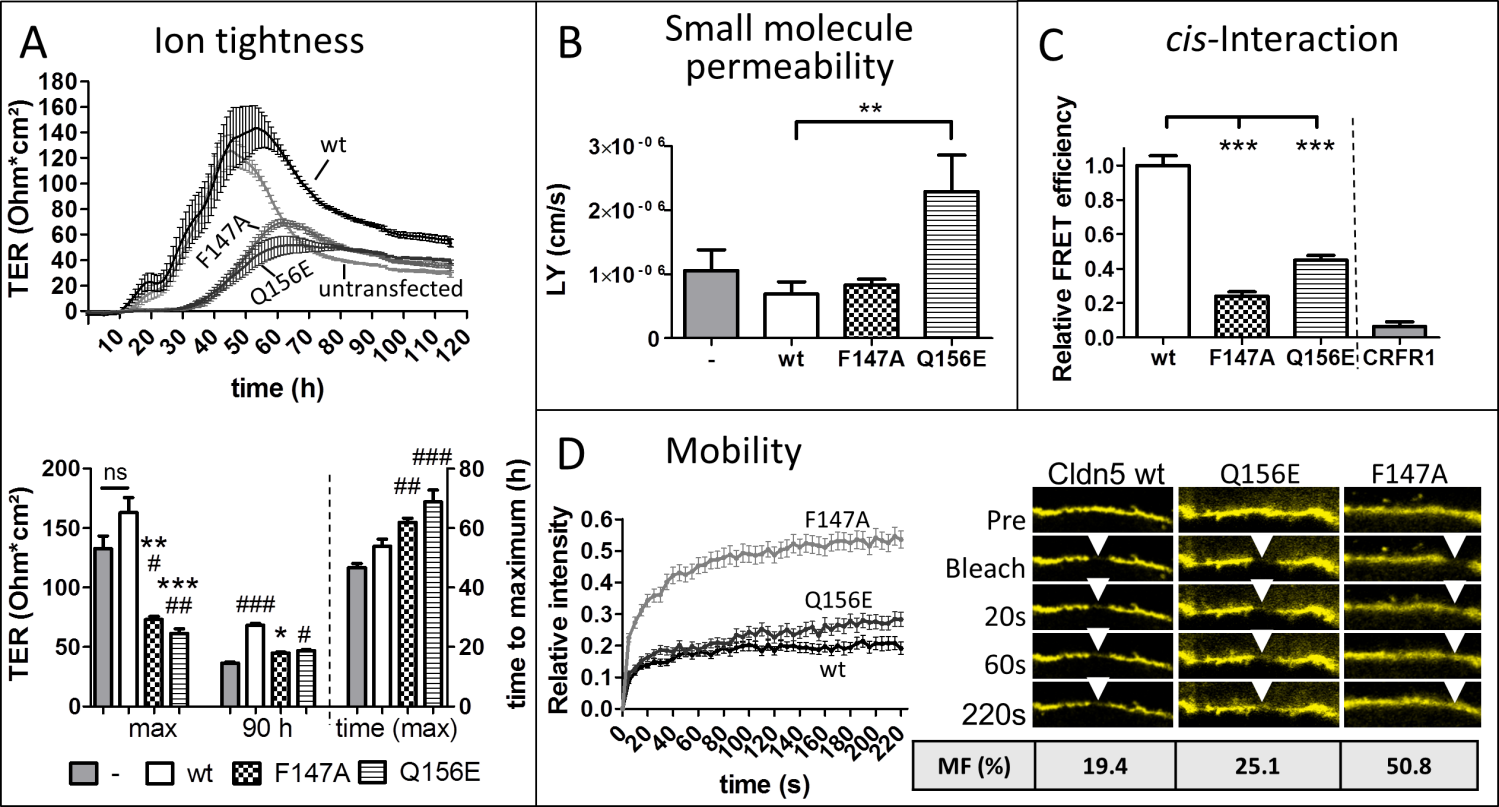
Mutation (_mut_) in the extracellular loop (ECL) 2 leads to decreased *cis*- and *trans*-interaction of claudins (Cldns). **A,B:** *trans*-Interactions assessed by transepithelial electrical resistance (TER) and permeability in filter-grown Madin-Darby canine kidney cells II expressing murine Cldn5_wild type_ (wt), Cldn5_F147A_, Cldn5_Q156E_ or untransfected (-). **A**: TER-values, n≥8. **B**: Permeability coefficient for lucifer yellow across the monolayer, n = 9. **C**: Homophilic *cis*-interaction between yellow fluorescent protein (YFP)/mTurquoise2 pairs of murine Cldn5_wt_ or Cldn5_mut_ was measured as fluorescence resonance energy transfer (FRET)-efficiency along the plasma membrane between transiently transfected HEK-293 cells. CRFR: Corticotropin-releasing factor receptor 1 (negative control). Values were normalized to Cldn5_wt_, n>25. **D:** Mobility of YFP-Cldn5 in the membrane measured by fluorescence recovery after photobleaching (FRAP). MF: mobile fraction, calculated from fitted curve. n>26. Mean±SEM, *p<0.05, **p<0.01, ***p<0.001, ns: not significant. Kruskal-Wallis test with Dunn’s post hoc test. Differences shown compared to Cldn5_wt_ transfected (*) or untransfected cells (#).

To elucidate the impact of Cldn-Cldn interactions on cross-over endocytosis, murine Cldn5 with amino acid (aa) substitutions in the ECL2 was transfected into MDCK-II cells. Substitutions Cldn5_F147A_ and Cldn5_Q156E_ were previously found to impair homophilic *trans*-interactions; *cis*-interactions as measured by fluorescence energy transfer (FRET) were not affected when coexpressed with wildtype (wt) Cldn5 [2]. After seeding, the transepithelial electrical resistance (TER) of MDCK-II cells developed a tightness curve which was characterized by a peak followed by a plateau. TER was increased in cells expressing Cldn5_wt_ compared to untransfected cells similar to earlier findings [22]. This increase was significant at the beginning of the plateau 90 min post seeding (Fig 2A, from 36.5±0.9 to 68.3±1. Ω·cm^2^). Cldn5_F147A_ and Cldn5_Q156E_ substituents showed lower TER values (44.9±0.8 and 47.9±0.9 Ω·cm^2^, respectively) than Cldn5_wt_ transfected cells 90 h post seeding and at the peak (untransfected: 132.7±10.7, wt: 163.0±12.7, F147A: 73.1±2.4, Q156E: 61.3±4.0 Ω·cm^2^). These reduced values indicate that the positions F147 and Q156 are involved in the paracellular tightening function of Cldn5. Moreover, the peaks reached by Cldn5_F147A_ and Cldn5_Q156E_ was significantly lower than the peak reached by untransfected cells, which indicates that Cldn5_F147A_ and Cldn5_Q156E_ interfere with the function of endogenous Cldns. There was also a significant delay in the peak for Cldn5_F147A_ and a still greater delay for Cldn5_Q156E_ (Fig 2A). The barrier permeability for lucifer yellow (457 Da) was nearly unchanged for Cldn5_F147A_ transfected cells, while transfection of Cldn5_Q156E_ resulted in a strong increase in the permeability compared to Cldn5_wt_-transfected cells (Fig 2B). That means Cldn5_F147A_ resulted in an opening for molecules smaller than 457 Da only.

FRET measurements revealed reduced *cis*-interactions for Cldn5_F147A_/Cldn5_F147A_, and for Cldn5_Q156E_/Cldn5_Q156E_. However, Cldn5_F147A_ was more severely affected (Fig 2C). The membrane mobility of Cldn5_Q156E_ was slightly enhanced compared to that of wt Cldn5, while Cldn5_F147A_ showed an increased mobility in the plasma membrane as measured by fluorescence recovery after photobleaching (FRAP). Cldn5 displayed a mobile fraction of 19%, Cldn5_Q156E_ 25%, and Cldn5_F147A_ 51% (Fig 2D). Cldn5_F147A_ and Cldn5_Q156E_ transfection led to lower peaks in TER measurement and, in case of Cldn5_Q156E_, to higher permeability compared to untransfected cells (Fig 2A). This suggests that Cldn5_F147A_ and Cldn5_Q156E_ are incorporated into the TJs and interfere with the function of endogenous Cldns.

### Impaired claudin-claudin interactions lead to reduced cross-over endocytosis

**Fig 3:**
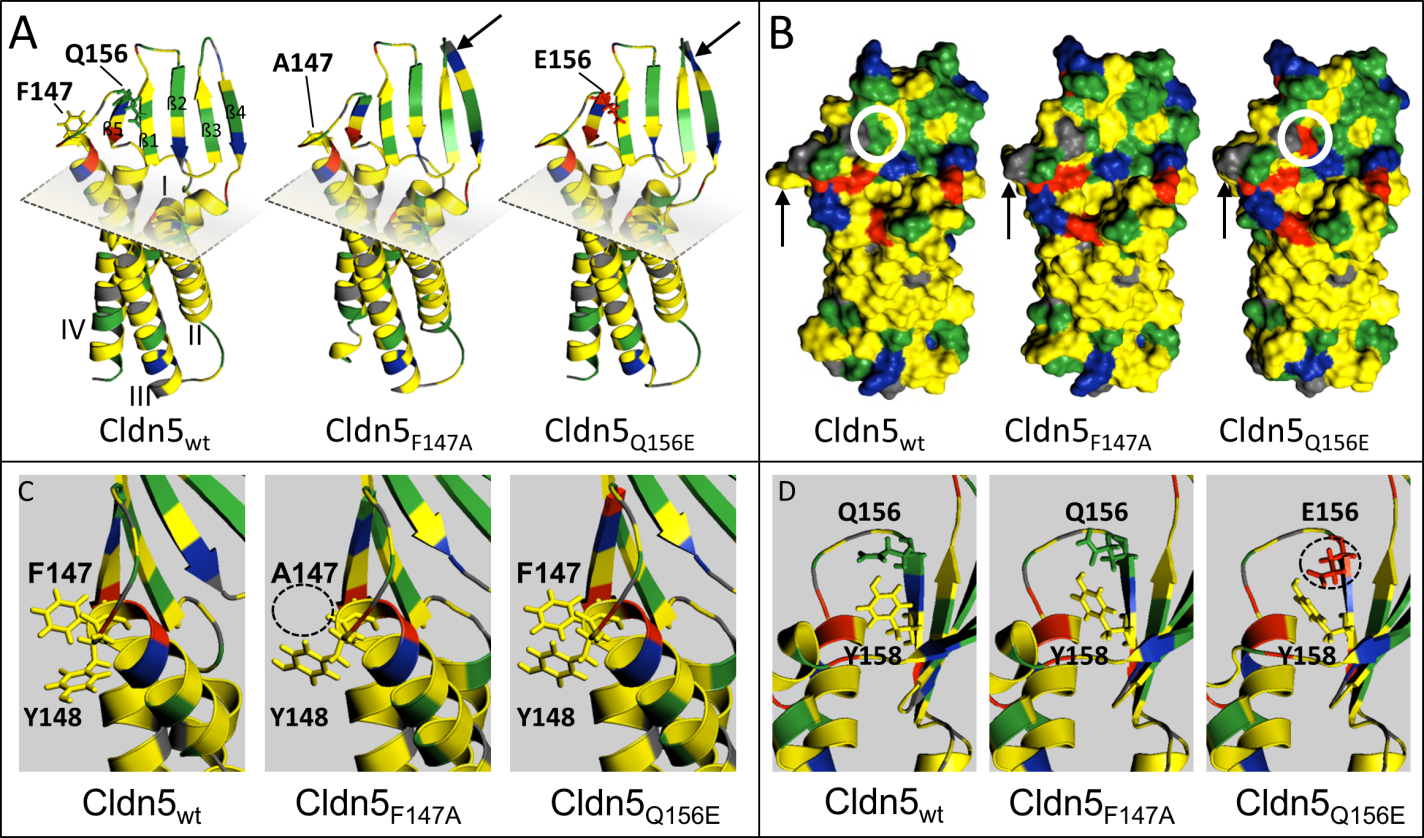
Amino acid (aa) substitutions F147A and Q156E lead to conformational changes in claudin-5 (Cldn5). **A-D:** I-TASSER modeling of murine Cldn5_wt_ and substitution variants Cldn5_F147A_ and Cldn5_Q156E_ (aa 1–190, extracellular loops and transmembrane domains). The modeled secondary structures of both substitutions were aligned with that of Cldn5_wt_ using PyMOL. Green: hydrophilic, yellow: hydrophobic, red: negatively charged, blue: positively charged, gray: neutral. **A**: Ribbon model. The four transmembrane helices (I–IV) and β-strands (β1–5) of the extracellular loops are labeled, as well as substituted aa F147 and Q156. Gray plane: outer plasma membrane surface. Arrows: elongation of β3 and β4. **B**: Surface model. Arrows: conformational changes in proximity to F147. White circle: surface charge introduced to binding domain for *trans*-interaction in Cldn5_Q156E_. **C,D:** Conformational changes in aromatic residues F147, Y148 and Y158 after aa substitutions. Circles: major changes.

Modeling with I-TASSER revealed slight conformational changes in the secondary structure of the aa-substituents, i.e. elongation of the third and fourth β-strand in the ECL1 (Fig 3A, arrows). In the surface model, changes in the binding interface became visible and are visualized by a white circle. Cldn5_Q156E_ led to a charged residue being introduced into the binding interface (Fig 3B, right), which explains the altered binding properties. In Cldn5_F147A_, only slight surface changes occurred (Fig 3B, middle), consistent with the unchanged barrier properties measured by TER (Fig 2A). However, in the models of Cldn5_F147A_ and Cldn5_Q156E_, the conformation of the aromatic side chains of Y148 and Y158 was altered (Fig 3C and 3D). F147 and Y148 are part of an aromatic binding interface involved in *trans*-interactions, Y158 is also involved in *cis*-interactions [2]. The conformational changes induced by the amino acid substitutions F147A and Q156E on Y148 could be responsible for the reduction of *cis*-interactions measured by FRET (Fig 2C).

**Fig 4:**
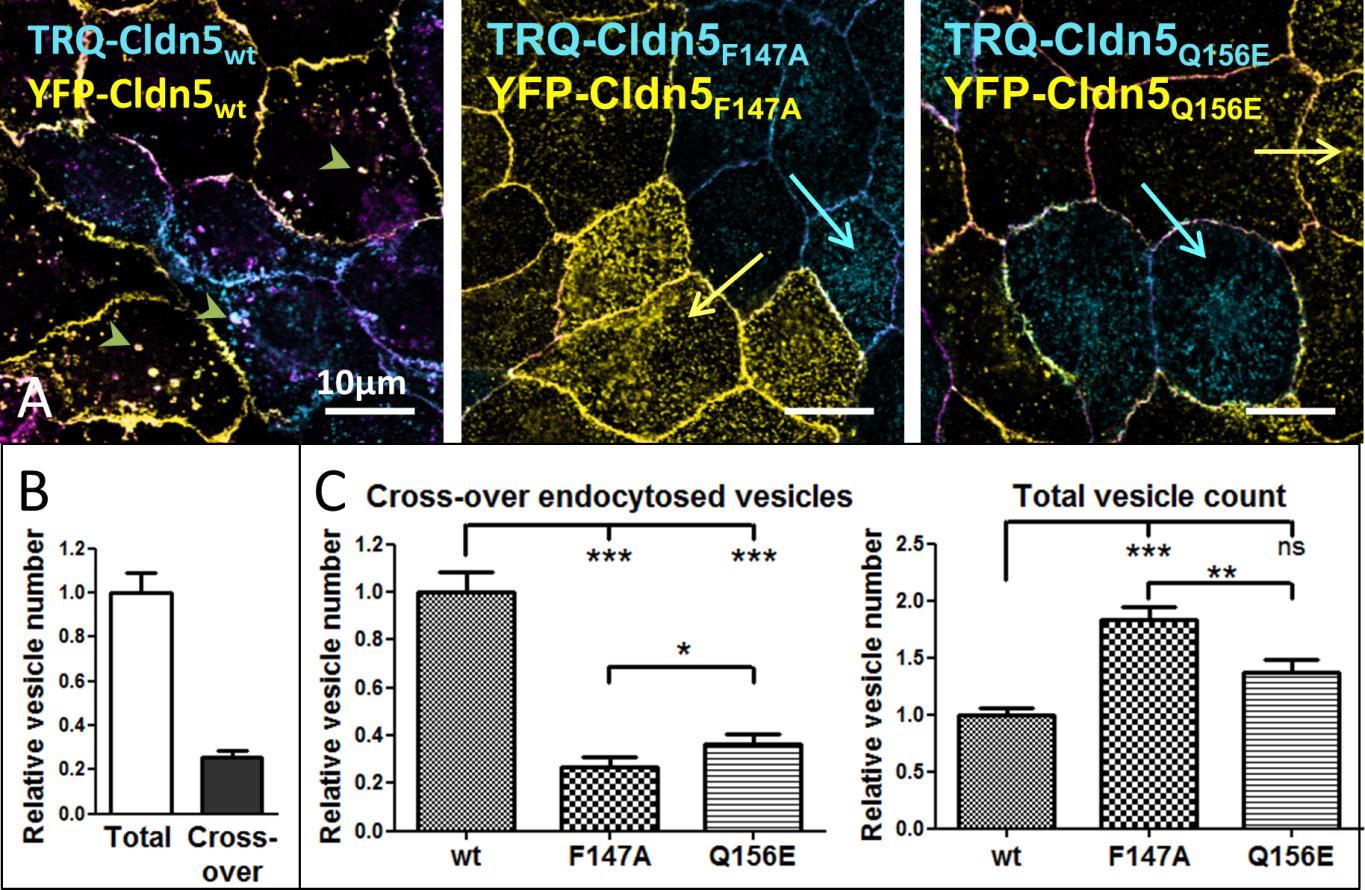
The extracellular loop 2 (ECL2) of claudin-5 (Cldn5) is involved in cross-over endocytosis. Murine Cldn5-mutants with an amino acid substitution in ECL2 fluorescently labelled with yellow fluorescent protein (YFP) or mTurquoise2 (TRQ) were expressed in Madin-Darby canine kidney cells II and grown in cocultures of monotransfected cells. **A**: Localization of Cldn5_wt_, Cldn5_F147A_ and Cldn5_Q156E_. Arrowheads: cross-over endocytosed vesicles (white color). Arrows: vesicles containing only one type of Cldn as indicated by the color of the arrow (not cross-over endocytosed). Purple: ZO-1 used as junctional marker. **B:** Vesicle numbers of Cldn_wt_. Relative number of cross-over endocytosed vesicles vs. total TRQ-Cldn5 vesicles in untreated cocultures; mean±SEM, n>100. **C**: Vesicle numbers normalized to Cldn5_wt_; mean±SEM, for each group n>70. *p<0.05, **p<0.01, ***p<0.001; one-way ANOVA.

When transfected into MDCK-II cells, Cldn5 is mainly located in the plasma membrane, but many vesicles can be found intracellularly (Fig 4A). Approximately 25% of these vesicles contained cross-over endocytosed Cldn5 (Fig 4B). Cldn5_F147A_ and Cldn5_Q156E_ were also located in the plasma membrane (Fig 4A) but there were more total intracellular vesicles as compared to Cldn5_wt_ (Fig 4A, arrows; Fig 4C, right) while the number of cross-over endocytosed vesicles was dramatically decreased (Fig 4A, arrowheads; Fig 4C, left). Cldn5_F147A_ expressing cells were more severely affected than Cldn5_Q156E_ expressing cells regarding vesicle numbers. Cldn5_F147A_ showed less cross-over endocytosed and more total vesicles than Cldn5_Q156E_ (Fig 4C). This elevated total vesicle count may reflect a higher rate of single membrane endocytosis and recycling. In the case of Cldn5_F147A_, vesicles seem to colocalize more frequently with Rab11 than wt Cldn5-containing vesicles (Panel A in S2 Fig). However, this was not observed for Rab4 (Panel B in S2 Fig).

### Cross-over endocytosis is affected by chlorpromazine, an inhibitor of the clathrin pathway, and filipin III, an inhibitor of the caveolin pathway

**Fig 5:**
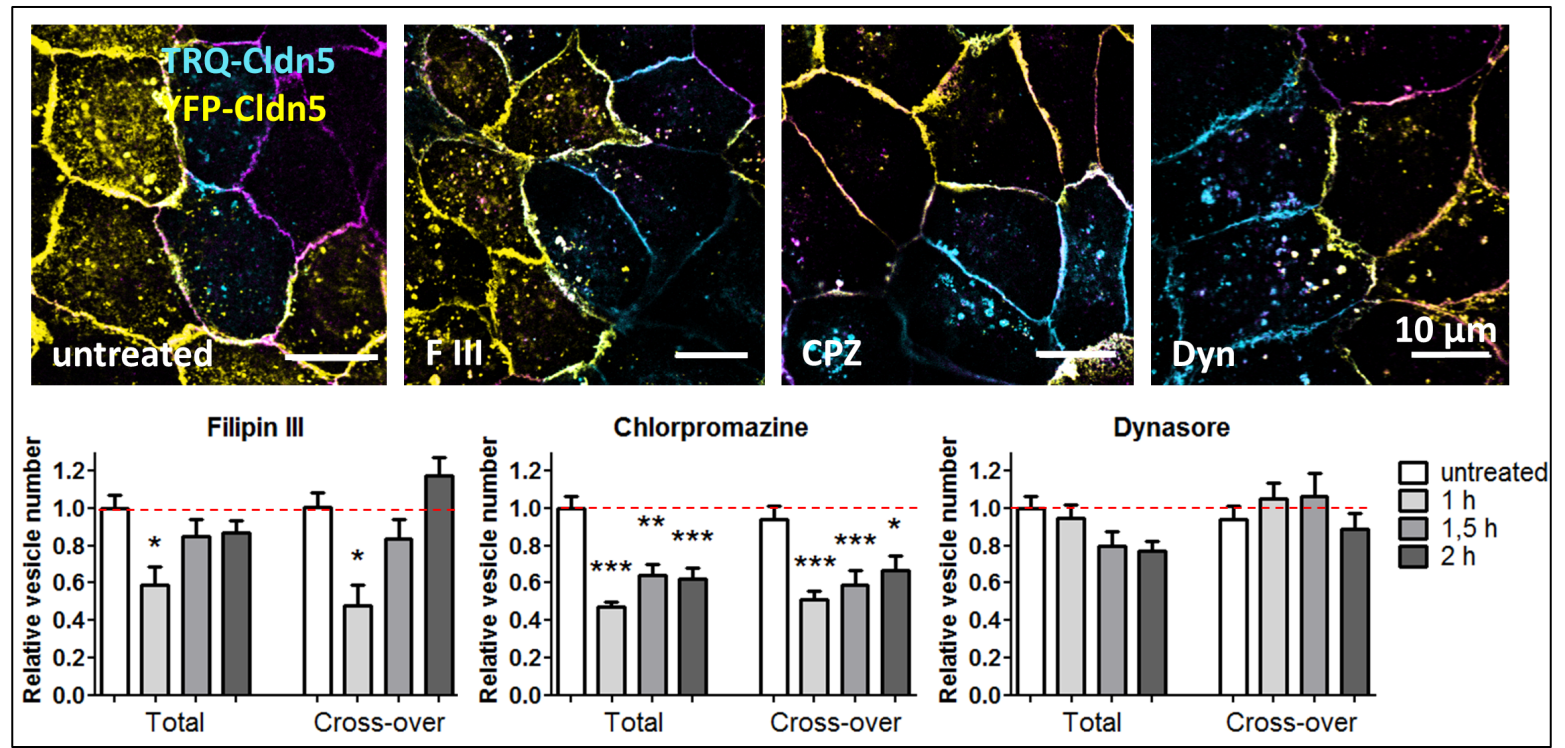
Fluorescently labeled claudin-5 (Cldn5) cross-over endocytosed via clathrin pathway. Cocultures of monotransfected Madin-Darby canine kidney cells II expressing Cldn5 fluorescently labeled with mTurquoise2 (TRQ) or yellow fluorescent protein (YFP). Treatment with inhibitors of caveolae-formation (filipin III, FIII; 2 h), clathrin-mediated endocytosis (chlorpromazine, CPZ; 1 h) and dynamin (dynasore, Dyn; 2 h). Purple: endogenous Cldn1. Relative numbers of TRQ-Cldn5 vesicles after treatment with inhibitors; mean±SEM, n>50. *p<0.05, **p<0.01, ***p<0.001, compared to the untreated control; one-way ANOVA.

MDCK-II cells were treated with inhibitors of various pathways of endocytosis (Fig 5). Vesicles containing TRQ-Cldn5 were counted in TRQ-Cldn5-transfected cells (total vesicle number) and in YFP-Cldn5-transfected cells (cross-over endocytosed vesicles). For total vesicles, a decrease was found after treatment with all inhibitors tested, however that with dynasore (Dyn) was a trend only (Fig 5). The strongest (approx. 50% reduction) and statistically significant effect was observed after treatment with chlorpromazine (CPZ), an inhibitor of clathrin adaptor protein 2 (AP-2) [25]. Inhibition of caveolae formation using filipin III (F3) [26] led to decreased vesicle numbers within 1 h after treatment, and inhibition of dynamin using Dyn [27] resulted in a non-significant reduction in vesicle numbers. For TRQ-Cldn5 vesicles cross-over endocytosed into YFP-Cldn5 transfected cells, a strong decrease in vesicle numbers was seen after treatment with CPZ and F3. F3 reduced the vesicle numbers transiently after 1 h of treatment, while CPZ led to a longer lasting reduction. Treatment with Dyn did not lead to a significant change in the number of cross-over endocytosed vesicles. A similar scenario is known from *D*. *melanogaster* during elongation of cell-cell contacts. Addition and local removal of adherence junctions was accompanied by accumulation of the clathrin machinery at the junctions, but Dyn inhibition had no effect on junctional remodeling [28].

### Lysosomal inhibitor chloroquine leads to accumulation of cross-over endocytosed claudins

**Fig 6:**
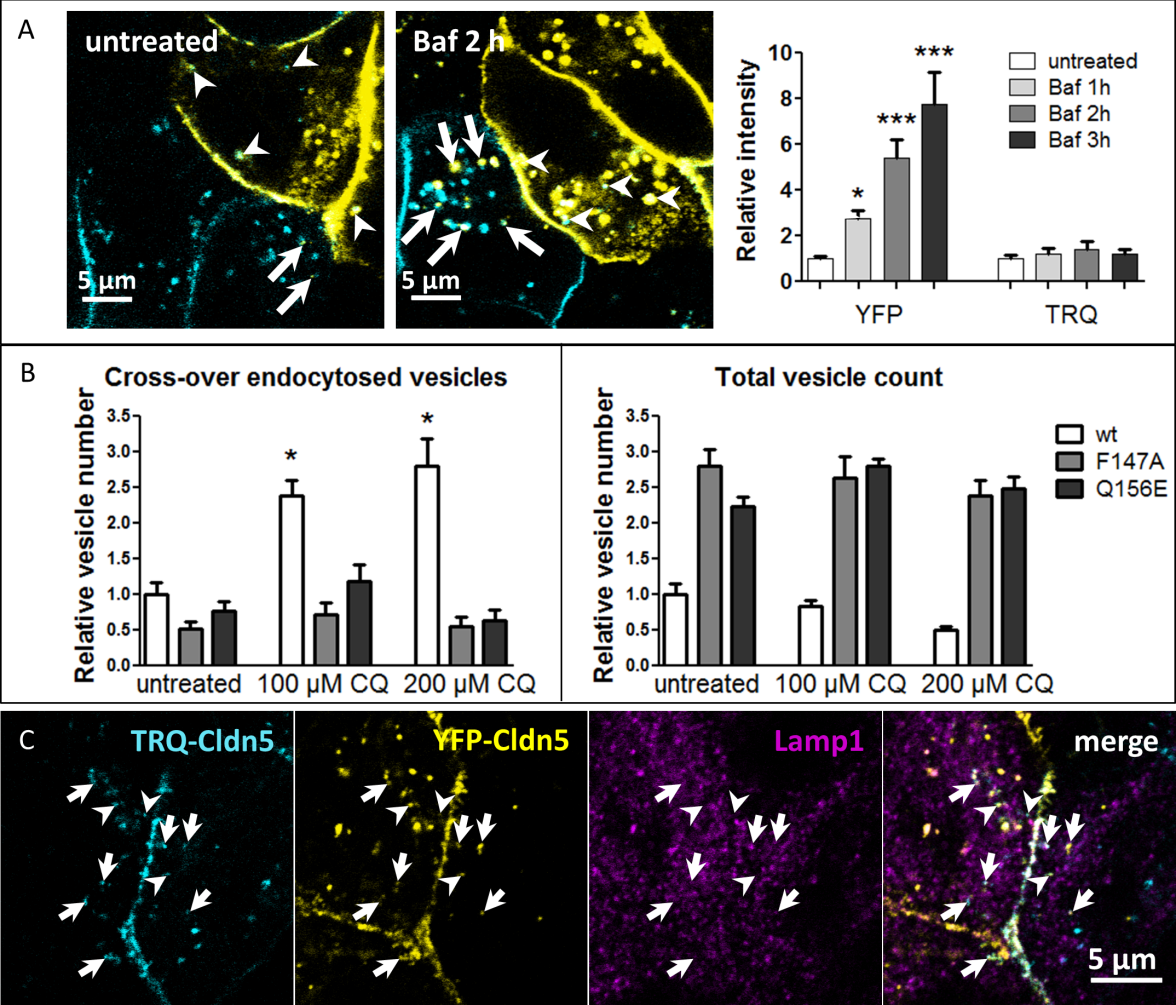
Cross-over endocytosed claudins (Cldns) are degraded via the lysosome. Cocultures of monotransfected Madin-Darby canine kidney cells II cells expressing Cldn fluorescently labeled with mTurquoise2 (TRQ) or yellow fluorescent protein (YFP). **A**: Live-cell imaging of TRQ-/YFP-Cldn5 vesicles after treatment with Bafilomycin A1 (Baf, 2 h, 100 nM). Arrows: cross-over endocytosed YFP-Cldn5. Arrowheads: cross-over endocytosed TRQ-Cldn5. Treatment with Baf rescues YFP-fluorescence (right side). Intensities were normalized to untreated values. For each group n>17. **B**: Increased vesicle numbers of TRQ-Cldn5 after 3 h of chloroquine (CQ) treatment (fixed samples). For each group n>20, CQ treated samples were tested against untreated. **C**: Colocalization of cross-over endocytosed Cldn5 with the lysosome marker lysosomal-associated membrane protein 1 (Lamp1). Arrows: vesicles containing TRQ-Cldn5, YFP-Cldn5 and Lamp1. Arrowheads: Lamp1-negative vesicles containing TRQ-Cldn5 and YFP-Cldn5. Mean±SEM, *p<0.05, ***p<0.001; one-way ANOVA.

In live cell imaging experiments, vesicles containing TRQ-Cldn were frequently observed in YFP-Cldn expressing cells; however, fewer YFP-Cldn positive vesicles were visible in TRQ-Cldn cells (Fig 6A, arrows). This observation may reflect the pH-sensitivity of YFP [29], if cross-over endocytosed Cldns are transported to cellular compartments with acidic environment, such as lysosomes. Treatment with Bafilomycin A1 (baf, an inhibitor of the V-H^+^-ATPase) which prevents endosomal acidification, led to a time-dependent increase of cross-over endocytosed YFP-Cldns (Fig 6A, arrows). After 3 h incubation with baf there was an 8-fold increase in cross-over endocytosed YFP-intensity, while there was no significant increase in TRQ-intensity.

In fixed samples, the number of cross-over endocytosed TRQ-Cldn5 vesicles was increased after inhibition of lysosomal degradation using chloroquine (CQ, a weak, cell permeable base that leads to alkalinization of lysosomes, Fig 6B). This effect was observed for Cldn5_wt_, as well as Cldn5_F147A_ and Cldn5_Q156E_ cells. However, much greater accumulation of cross-over endocytosed vesicles occurred for Cldn5_wt_ than for Cldn5_F147A_ and Cldn5_Q156E_ (Fig 6B, left). This accumulation supports the involvement of the lysosomal degradation pathway for cross-over endocytosed Cldn5 and confirms that there is less cross-over endocytosis for interaction-defective Cldn5_mut_. In untreated MDCK-II cells, cross-over endocytosed Cldn5 was found in vesicles containing the lysosomal marker lysosomal-associated membrane protein 1 (Lamp1) (Fig 6C), which confirms the contribution of the lysosome in the degradation of cross-over endocytosed Cldns. However, there were also Lamp1-negative vesicles containing cross-over endocytosed Cldn5, which indicates an intermediate step in the trafficking of these vesicles.

### Cross-over endocytosed vesicles colocalize with autophagosomal markers

**Fig 7:**
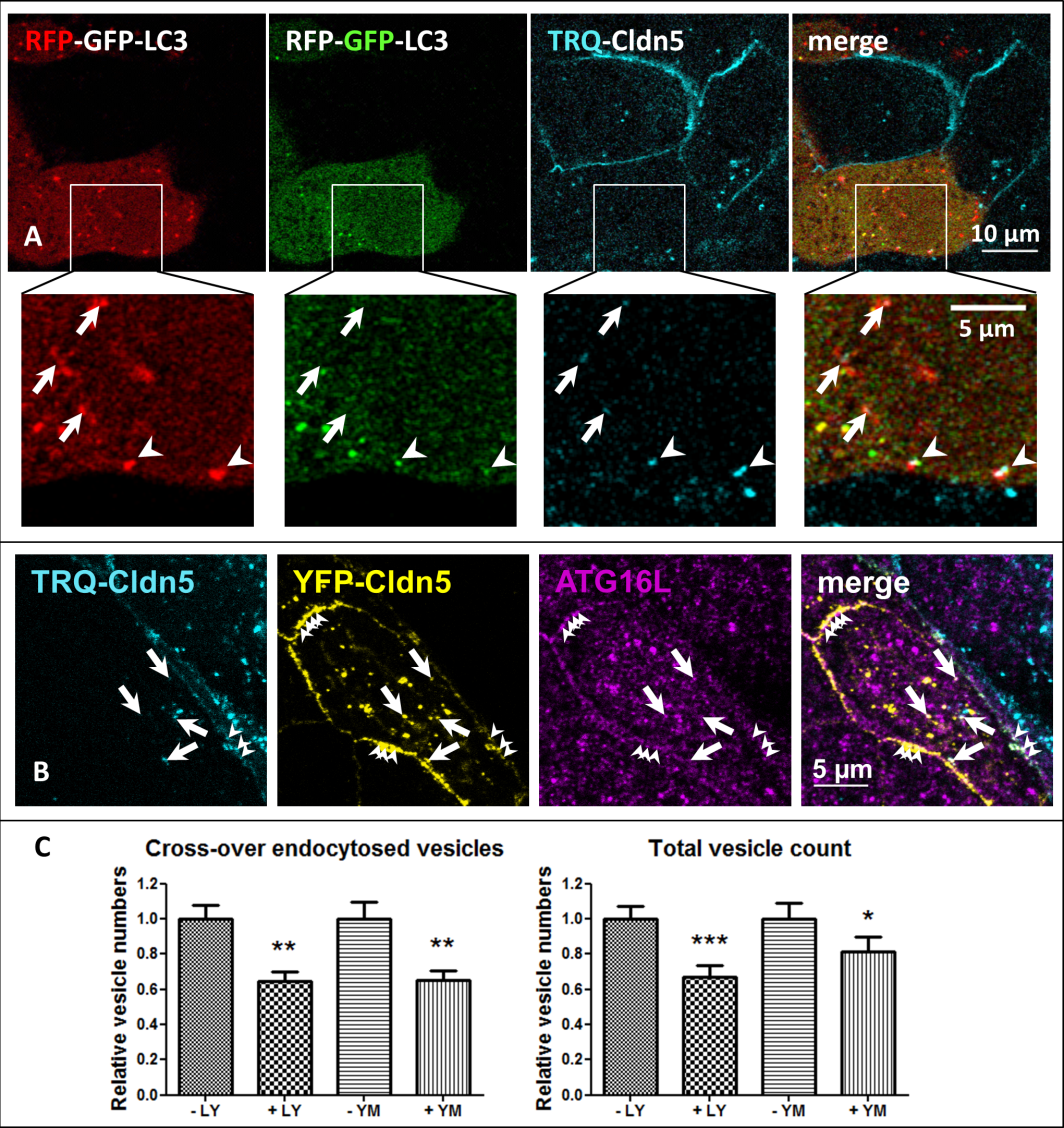
Autophagic machinery is involved in cross-over endocytosis. **A**: Cocultures of mTurquoise2 (TRQ)-Cldn5 or LC3 labeled with red and green fluorescent protein (RFP-GFP-LC3) mono-transfected in Madin-Darby canine kidney cells II (MDCK-II). Arrows: Cross-over endocytosed TRQ-Cldn5 colocalizes with RFP (in autolysosomes as RFP fluorescence is not affected within the acidic environment of autolysosomes). Arrowheads: TRQ-Cldn5 colocalizes with GFP and RFP (in neutral autophagosomes). **B, C:** Cocultures of monotransfected MDCK-II-cells expressing either yellow fluorescent protein (YFP)-Cldn5 or TRQ-Cldn5. **B:** Immunofluorescent staining against autophagy related protein 16L (ATG16L). Arrows: ATG16L colocalizes with cross-over endocytosed Cldn5. Arrowheads: ATG16L colocalizes with Cldn5 at the plasma membrane. **C**: Decrease in vesicle numbers of TRQ-Cldn5 in fixed samples after incubation with PI3K inhibitor LY294002 (2.5 h) and PIKfyve inhibitor YM-201636 (1 μM, 3 h). Mean±SEM, for each group n>50. *p<0.05, **p<0.01, ***p<0.001; two-sided t test.

Next, the prelysosomal Lamp1-negative vesicles containing cross-over endocytosed Cldn5 were further characterized. The endocytosis of integral membrane proteins from two neighboring cell membranes indicates a double-membrane morphology of the vesicle, reminiscent of the structure of autophagosomes. To test the involvement of the autophagosome, cocultured cells expressing either TRQ-Cld5 or the autophagosomal marker microtubule-associated protein 1A/1B-light chain 3 (LC3) [30], tagged with red and green fluorescent protein (RFP-GFP-LC3), were observed under live conditions. This construct monitors autophagic flux: in the cytoplasm and non-acidic autophagosomes both fluorophores of the tandem LC3 are visible, while in compartments with low pH, such as autolysosomes, only RFP is functional [31], as GFP is quenched at low pH [32]. Cross-over endocytosed TRQ-Cldn5 vesicles in RFP-GFP-LC3-transfected cells frequently colocalized with the RFP-signal alone (Fig 7A, arrows), and with both RFP and GFP, prominently near the plasma membrane (Fig 7A, arrowheads). Colocalization with GFP indicates the presence of TRQ-Cldn5 in autophagosomes before acidification, i.e. before fusion with the lysosome. Immunostaining of fixed YFP-Cldn5/TRQ-Cldn5 cocultures of monotransfected MDCK-II cells against autophagy related protein 16L (ATG16L) revealed a colocalization with cross-over endocytosed Cldn5 (Fig 7B, arrows) as well as with Cldn5 at the cell membrane (arrowheads).

Phosphoinositide 3-kinase (PI3K) and phosphatidylinositol-3-phosphate 5-kinase (PIKfyve) have been implicated in the formation of autophagic vesicles [33, 34]. Inhibition of PI3K using LY294002 or of PIKfyve using YM-201636 resulted in a reduction in cross-over endocytosed vesicles by 35%, which further supports involvement of the autophagic machinery in the cross-over endocytosis of Cldns.

### Super-resolution microscopy reveals double-membrane structure of cross-over endocytosed vesicles

**Fig 8:**
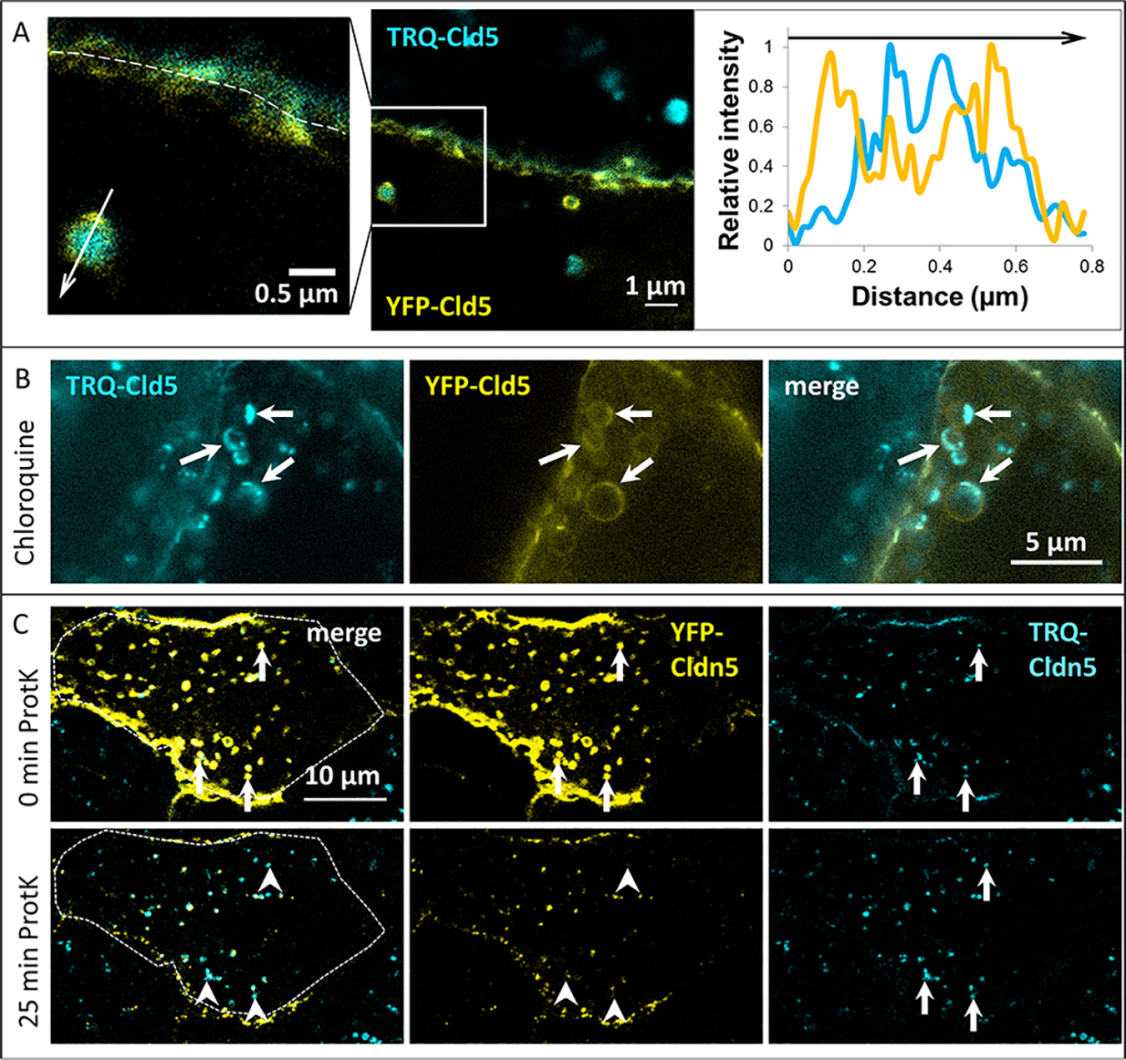
Cross-over endocytosed claudins (Cldns) are located in double-membrane vesicles. Cocultures of monotransfected Madin-Darby canine kidney cells II expressing Cldn5 fluorescently labeled with mTurquoise2 (TRQ) or yellow fluorescent protein (YFP). **A**: Live cell stimulated emission depletion (STED) images of untreated cells. Dashed line: cell-cell contact between a YFP-Cldn5 and a TRQ-Cldn5 expressing cell. Intensity profiles (right panel) of YFP- and TRQ-distribution along the arrow are normalized to the maximum intensity of each fluorophore. **B**: STED images of vesicles after 1 h treatment with the lysosome inhibitor chloroquine (CQ, 100 μM). Arrows: accumulated cross-over endocytosed Cldn5 in enlarged lysosomes. **C**: Fluorescence protease protection assay confirming outward directed YFP-Cldn5 and inward directed cross-over endocytosed TRQ-Cldn5 in double-membranous vesicles observed in YFP-Cldn5 transfected cells. Cells were recorded for 25 min after permeabilization of the membrane and addition of proteinase K (ProtK). Arrows: vesicles containing cross-over endocytosed TRQ-Cldn5. Arrowheads: reduced YFP-signal after ProtK digestion.

Stimulated emission depletion (STED) microscopy was applied to visualize the membrane topology of cross-over endocytosed Cldns in endosomes of subdiffraction size in living cells. Imaging cocultured TRQ-Cldn5 and YFP-Cldn5 transfected cells using gated STED microscopy with approximately 50 nm resolution [35] showed clear separation of cross-over endocytosed Cldns and Cldns expressed by the observed cell within the same vesicle (Fig 8A). In untreated cells, vesicles containing TRQ-Cldn5 were measured in YFP-Cldn5 transfected cells. The intensity profile across the vesicle clearly showed that YFP was distributed in the outer membrane of the vesicle, while cross-over endocytosed TRQ-Cldn5 was located inside the vesicle (Fig 8A, right). After treatment with the lysosomal inhibitor CQ, larger YFP-Cldn5-containing structures became visible, with accumulation of TRQ toward the side of the vesicle (Fig 8B, arrows). As CQ leads to the dilatation of lysosomes [36], the outer membrane of cross-over endocytosed vesicles corresponds to the lysosomal membrane. The diameter of the outer membrane of cross-over endocytosed vesicles in untreated cells ranged between 400 and 900 nm (n = 9), while vesicles in CQ-treated cells were larger (0.6 to 2 μm, n = 8).

**Fig 9:**
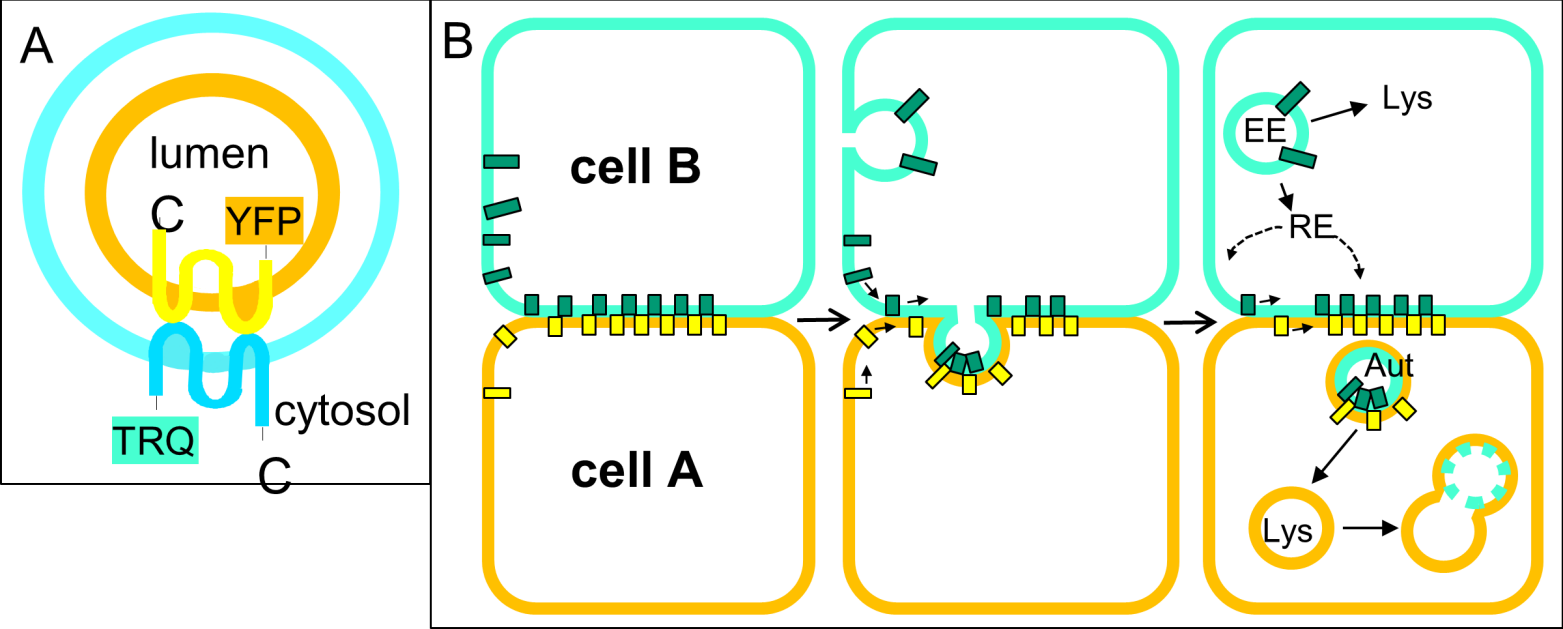
Mechanism of cross-over endocytosis. **A**: Structure of tight junction (TJ) derived vesicle. After endocytosis from the TJ, a double-membrane vesicle contains claudins (Cldns) from both cells. The fluorophores attached to the N-terminus of the cross-over endocytosed Cldn is directed toward the lumen of the vesicle. **B**: In cross-over endocytosis, strong intercellular Cldn-interactions are not released. The TJ complex is internalized into an autophagosome-like vesicle and delivered to the lysosome where the inner membrane (which is derived from the neighboring cell) is digested. EE: early endosome; RE: recycling endosome; Lys: lysosome; Aut: autophagosome. Blue and yellow rectangles: Cldn-molecules.

The results observed by STED microscopy were confirmed by a fluorescence proteinase protection (FPP) assay. In this approach, the plasma membrane (but not organelle membranes) of live cells was selectively permeabilized and proteinase K was added to the cells. Fluorophores directed toward the cytoplasm were digested, while those contained inside vesicles were protected from degradation. In YFP-Cldn5 transfected cells containing cross-over endocytosed TRQ-Cldn5, only YFP-Cldn5 was noticeably digested after 25 min of exposure to proteinase K (Fig 8C, arrows), while TRQ-Cldn5 was not. This confirms the differential orientation of Cldn termini. While fluorophores from neighboring cells were located inside the vesicle and protected from digestion, fluorophores of Cldns derived from the observed cell were oriented toward the cytosol (Fig 9A).

## Discussion

In this study, a direct involvement of the autophagic machinery in TJ-remodeling is demonstrated, and for the first time the presence of Cldns is shown in large double membrane vesicles. Our results confirm a type of endocytosis of Cldns, where TJ-associated Cldn-oligomers are cross-over endocytosed into the neighboring cell in double-membrane vesicles in a clathrin- and caveolin-dependent manner. Moreover, cross-over endocytosis depends on Cldn interactions.

The ECLs of Cldns strongly interact with Cldns of neighboring cells; interactions between Cldn1/Cldn1, Cldn5/Cldn5 or Cldn1/Cldn5 have been shown to reach affinity values in the nanomolar range [18]. This very high affinity leads to strong adhesion between cells and effectively hinders their separation. The oligomerization of Cldns across the paracellular cleft is mediated by their ECLs [2, 22]. Amino acid exchange in the ECL2 of Cldn5 in the positions F147 and Q156 leads to decreased *trans*-interactions, but was thought not to influence *cis*-interactions when Cldn5_F147A_ or Cldn5_Q156E_ was measured against Cldn5_wt_ [2]. However, we now find that *cis*-interactions were compromised in a FRET assay, when Cldn5_F147A_/Cldn5_F147A_ or Cldn5_Q156E_/Cldn5_Q156E_ pairs were analyzed. The earlier measurements for substitution/wt pairs show that impaired *cis*-interactions can be rescued by association with other Cldns. This means that other parts of the molecule compensate for the deficit in binding, probably the ECL1 which is well known to contribute to paracellular tightness [37].

In MDCK-II cells endogenous Cldn1 is expressed, which is able to interact strongly with Cldn5 in *cis* and *trans* [17]. However, the transfection of Cldn5_F147A_ or Cldn5_Q156E_ interferes with barrier properties compared to untransfected cells, indicating that the endogenously expressed Cldns were not able to rescue the defects in *trans*-interactions. Interestingly, Cldn5_F147A_ showed greatly increased mobility within the membrane, and *cis*-interactions and cross-over endocytosis are decreased more strongly than for Cldn5_Q156E_. In contrast, the permeability of the cell layer is strongly increased in Cldn5_Q156E_-expressing cells, while *cis*-interactions are moderately affected and mobility is not affected. This suggests that the mobility of Cldns in the membrane depends on Cldn-Cldn-interactions, but does not necessarily have an impact on barrier tightness.

Permeability changes demonstrate that the substituents are incorporated into the TJs however they show less cross-over endocytosis than Cldn5_wt_. This is evidence that only the highly adhesive sections of the neighboring membrane are pulled into the neighboring cells. This adhesiveness is proven by testing the permeability, an equivalent of *trans*-associations closing the intercellular cleft. Our results also suggest a correlation of *cis*-interactions and cross-over endocytosis, as the substituent F147A, which exhibits weaker *cis*-interactions than Q156E, is also less cross-over endocytosed than Q156E. Colocalization of endogenous Cldn1, Cldn2, Cldn7 and occludin reveals that this mechanism of endocytosis is not targeting specific types of TJs-proteins for endocytosis, but rather specific parts of the TJs in which the proteins are complexed.

After endocytosis, Cldns can either be recycled back to the plasma membrane, or targeted for degradation [38]. Our experiments show that cross-over endocytosed Cldns are delivered to the lysosome using the autophagosomal machinery. Recently, it has been reported that the plasma membrane can be a membrane donor for autophagosome formation: Atg16L, a protein involved in the initiation of autophagosome formation, interacts with clathrin heavy chain to form autophagosomal precursors at the plasma membrane [39]. We find Atg16L to colocalize with Cldn5 in cross-over endocytosed vesicles as well as at the plasma membrane. The presence of GFP-LC3, which indicates an early autophagosome, in cross-over endocytosed vesicles confirms a role of the autophagosomal machinery in the induction of TJ-remodeling. This is further supported by the reduction of cross-over endocytosis after inhibition of PI3K and PIKfyve, kinases involved in autophagosome biogenesis [33, 34]. Autophagy plays a crucial role in maintaining cellular homeostasis, by the removal of large particles and aggregates from the cytosol. The phagophore (precursor of the autophagosome) is derived from the single-membranous endoplasmic reticulum, among other sources [40]. It elongates and encloses intracellular particles, thereby forming typical double-membrane vesicles that fuse with lysosomes to deliver their cargo for degradation [41]. In contrast, cross-over endocytosis does not seem to involve such single-membranous stages as the phagophore. The molecular basis of the biogenesis of autophagosome-like vesicles that do not develop from a phagophore remains elusive. However, the effects of PI3K and PIKfyve inhibition suggest that phosphatidylinositol phosphates are involved in the regulation of this mechanism, possibly by specifying the part of the TJ targeted for endocytosis.

This mechanism differs from conventional endocytosis in a critical way: instead of a single membrane being endocytosed, a patch of the TJ containing two cell membranes is removed as a double-membrane vesicle into one of the cells. In super-resolution images, clear separation of the Cldns from both cells is visible within the vesicles, which indicates that both plasma membranes are incorporated. Cldns derived from the cell containing the vesicle are directed toward the cytosol, while the termini of cross-over endocytosed Cldns of the neighboring cell are directed toward the lumen of the vesicle (Fig 9A). In a fluorescence protease protection (FPP) assay, cross-over endocytosed TRQ-mCld5 accordingly was protected from digestion, as only fluorophores in the cytosol are accessible to the proteinase. The luminal position of cross-over endocytosed Cldn is also supported by the disappearance of the cross-over endocytosed YFP-signal. As YFP is sensitive to an acidic environment [29], its loss of fluorescence after cross-over endocytosis is due to the localization of the fluorophore inside the acidic lysosomal compartment. Accordingly, inhibition of lysosomal acidification by bafilomycin rescued YFP-fluorescence in our experiments. The colocalization of cross-over endocytosed Cldns with the autophagosomal marker LC3 might be interpreted as engulfment of the cross-over endocytosed vesicle by an autophagosome. However, the FPP assay implies that this is not the case, as both fluorophores would be protected from digestion if surrounded by an autophagosomal membrane. This conclusion is supported by the finding that the distribution cross-over endocytosed TRQ-Cldn5 signal is not affected by inhibition of lysosome function by chloroquine, while the outer membrane containing YFP-Cldn5 from the same cell grows in size (Fig 8B). Double membrane vesicles have been described in electron microscopy and assumed to contain Cldns. However, the presence of Cldns in these vesicles was not confirmed so far [19]. Our results now prove this assumption.

Inhibition of dynamin does not lead to a reduction in cross-over endocytosis, however inhibition of clathrin/AP-2-interaction and cholesterol depletion (filipin III treatment) does. Therefore scission of the nascent cross-over vesicle at the plasma membrane does not depend on dynamin, but the interaction with clathrin/AP-2 and caveolin/cholesterol has an impact on cross-over endocytosis. A similar scenario is known from in *Drosophila* during the elongation of cell-cell contacts. Addition and local removal of adherence junctions is accompanied by an accumulation of the clathrin machinery at the junctions but a dynamin inhibition has no effect on junctional remodeling [28]. Consequently further dynamin-independent mechanisms have to be considered. Thus, chlorpromazine causes relocation of clathrin and AP-2 from coated pits to endosomes [25]. However, the interaction of clathrin and AP-2 with ATG16L at the plasma membrane regulates the initiation of autophagy [39]. In this manner the clathrin pathway connects autophagy with cross-over endocytosis. The role of the caveolin pathway in autophagy is also documented: Cholesterol depletion leads to the induction of autophagy [42], as does caveolin knockdown [43]. Filipin III binds to cholesterol in the membrane and inhibits the interactions between cholesterol and caveolin, leading to inhibition of caveolae formation [26]. In addition to inhibition of endocytosis, filipin III can therefore reduce the number of cross-over endocytosed vesicles by increasing autophagic clearance of cross-over endocytosed Cldns.

Cross-over endocytosed vesicles contain various Cldn-subtypes as well as occludin. These are rather substantial and unspecific changes in TJ morphology, whereas the removal of specific Cldns from the TJ without eliminating the barrier function must be a strictly regulated process. Under nitric oxide stress, Cldn5 has recently been shown to be delivered for autolysosomal degradation in a caveolin-mediated process [44]. In the intestine autophagy has been shown to enhance barrier function by specifically down-regulating Cldn2 [45]. However, our results indicate, that the specificity of this down-regulation may not be achieved through selective internalization of a specific Cldn subtype, but rather by increased incorporation of other Cldns into the TJs after endocytosis of Cldn5- or Cldn2-rich TJ sections, respectively (Fig 9B). In addition, Cldn5 can be degraded by the proteasome pathway following ubiquitination, but also by an ubiquitin-independent mechanism [13], which still need to be clarified with respect to cross-over endocytosis.

The targeting of Cldns via a clathrin-dependent mechanism shares similarities with the internalization of some gap junction proteins. Connexin 31.1 can also be targeted by clathrin for degradation, via autophagy, in cell lines of non-small cell lung cancer [46]. For other connexins, the existence of small double-membrane vesicles has been proposed in cervix carcinoma cells [47]. In MDCK-II cells, cross-over endocytosed Cldn1 and -3 have been suggested to be involved in wound healing [19]. For adherence junctions, cadherin-dependent uptake of cell material from the neighboring cells has been described in fibroblasts [48]. This suggests a general mechanism for the endocytosis of transmembrane proteins that feature very strong affinity to proteins in neighboring cell membranes, such as Cldns [18]. This is further supported by the finding that, at the neuron-to-glia interface, the transmembrane EphB2 receptor is also cross-over endocytosed from neurons to neighboring glia cells [49].

In summary, our data illustrate the mechanism of TJ remodeling involving autophagy, where strongly adhering Cldns (via their extracellular loops) are not separated before endocytosis but internalized in an autophagosome-like double membrane vesicle (Fig 9B). In this process, both the interacting proteins and membranes from two neighboring cells are cross-over endocytosed into either cell. This vesicle then fuses with the lysosome, and the inner membrane (i.e. the membrane derived from a neighboring cell) is digested while the outer membrane becomes part of the lysosomal membrane. Cldn molecules not incorporated in the TJs, e.g. in the apical membrane, would be available for conventional endocytosis via the endosomal pathway (Fig 9B), while this is not possible for double membrane vesicles. Reports on other junctional proteins and transmembrane receptors support the assumption of a general mechanism for reorganizing cell-cell contacts. Collectively, the findings help to understand the highly dynamic behavior of cell-cell contacts, and demonstrate that autophagy is not only a pathological phenomenon but also of physiological relevance in cell-cell interaction across various cell types.

## Supporting information

S1 Fig Endogenous claudins (Cldns) and occluding (Occl) are present in cross-over endocytosed vesicles. Cocultures of monotransfected MDCK-II cells expressing either TRQ-Cldn5 or YFP-Cldn5 were immunostained against endogenous TJ proteins. Cross-over endocytosed vesicles contained Cldn2 and Cldn7 (upper and middle panel, arrows). Occl was found in some cross-over endocytosed vesicles (lower panel, arrows), some cross-over endocytosed vesicles were negative for Occl (arrowheads).
(TIF)

S2 Fig Cross-over endocytosed vesicles may colocalize with recycling vesicles. **A**: For Rab4, there was no considerable difference observed between Cldn5-expressing cells and Cldn5_F147A_ expressing cells. **B**: For Rab11, a slightly higher signal intensity was detected for Cldn5_F147A_-expressing cells (inset) compared to Cldn5-expressing cells. Cocultures of Madin-Darby canine kidney cells (line II) transfected either with TRQ-Cldn5/YFP-Cldn5 or TRQ-Cldn5_F147A_/YFP-Cldn5_F147A_ were immunostained against endogenous Rab proteins. Rab4 and Rab11, used as recycling markers, appeared to colocalize more frequently with non cross-over endocytosed Cldn (arrowheads), although some colocalization with cross-over endocytosed Cldn5 occurred (arrows). Most cross-over endocytosed vesicles did not contain Rab4 or Rab11 (open arrows). Cldn, claudin; TRQ, mTurquoise2 fluorescent protein; YFP, yellow fluorescent protein.
(TIF)
