# Supplementary figures and images for: Cross-over endocytosis of claudins is mediated by interactions via their extracellular loops
Source: PLoS One. 2017 Aug 15;12(8):e0182106. doi: 10.1371/journal.pone.0182106 (PMC5557494; doi:10.1371/journal.pone.0182106)

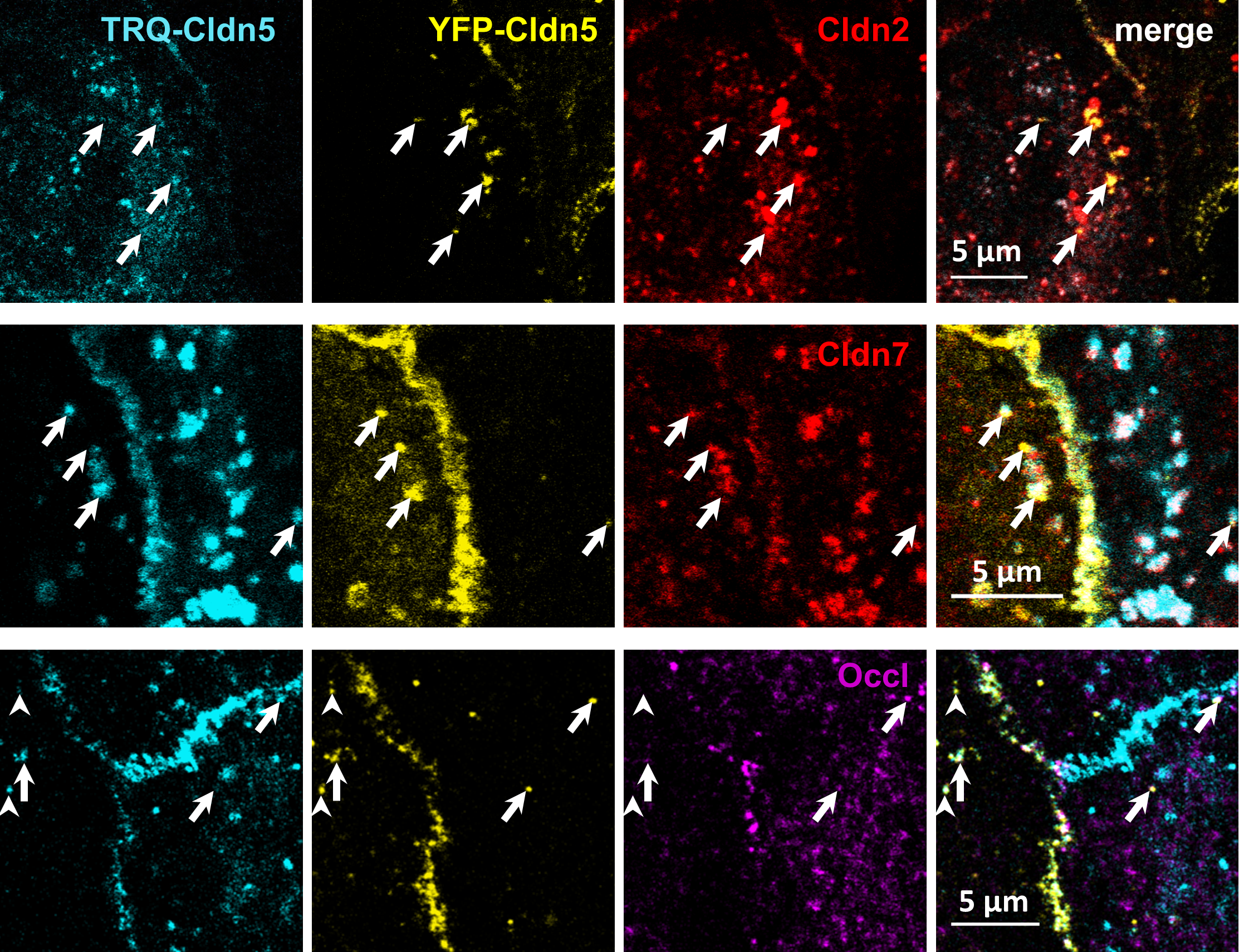

Supplement: S1 Fig — Cocultures of monotransfected MDCK-II cells expressing either TRQ-Cldn5 or YFP-Cldn5 were immunostained against endogenous TJ proteins. Cross-over endocytosed vesicles contained Cldn2 and Cldn7 (upper and middle panel, arrows). Occl was found in some cross-over endocytosed vesicles (lower panel, arrows), some cross-over endocytosed vesicles were negative for Occl (arrowheads). (TIF) [file pone.0182106.s001.tif]

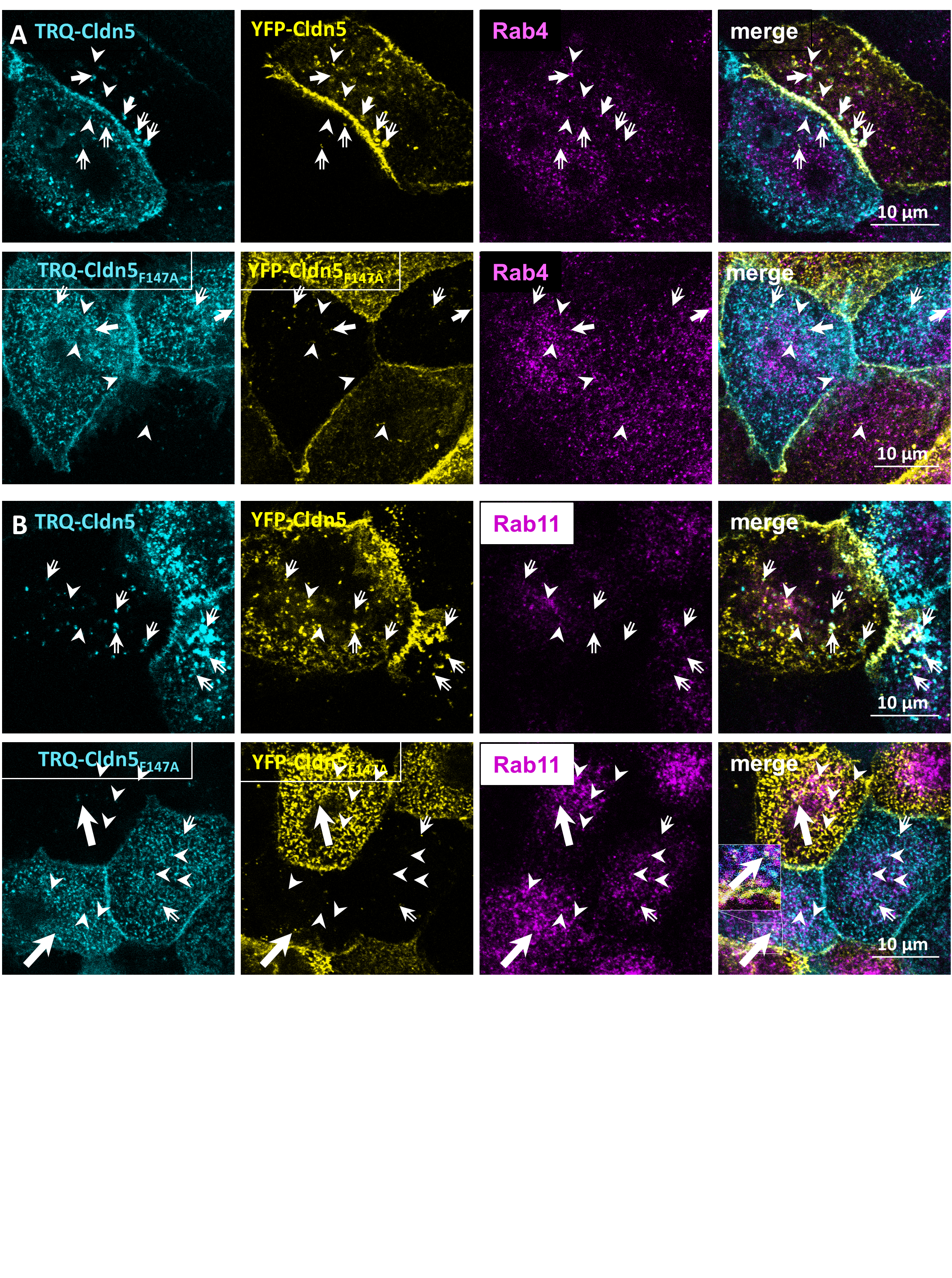

Supplement: S2 Fig — A: For Rab4, there was no considerable difference observed between Cldn5-expressing cells and Cldn5F147A expressing cells. B: For Rab11, a slightly higher signal intensity was detected for Cldn5F147A-expressing cells (inset) compared to Cldn5-expressing cells. Cocultures of Madin-Darby canine kidney cells (line II) transfected either with TRQ-Cldn5/YFP-Cldn5 or TRQ-Cldn5F147A/YFP-Cldn5F147A were immunostained against endogenous Rab proteins. Rab4 and Rab11, used as recycling markers, appeared to colocalize more frequently with non cross-over endocytosed Cldn (arrowheads), although some colocalization with cross-over endocytosed Cldn5 occurred (arrows). Most cross-over endocytosed vesicles did not contain Rab4 or Rab11 (open arrows). Cldn, claudin; TRQ, mTurquoise2 fluorescent protein; YFP, yellow fluorescent protein. (TIF) [file pone.0182106.s002.tif]
